# Review of Rare Earth Elements as Fertilizers and Feed Additives: A Knowledge Gap Analysis

**DOI:** 10.1007/s00244-020-00773-4

**Published:** 2020-11-03

**Authors:** Franca Tommasi, Philippe J. Thomas, Giovanni Pagano, Genevieve A. Perono, Rahime Oral, Daniel M. Lyons, Maria Toscanesi, Marco Trifuoggi

**Affiliations:** 1 Department of Biology, “Aldo Moro” Bari University, 70125 Bari, Italy; 2 Environment and Climate Change Canada, Science and Technology Branch, National Wildlife Research Center – Carleton University, Ottawa, ON K1A 0H3 Canada; 3 Department of Chemical Sciences, Federico II Naples University, 80126 Naples, Italy; 4 Department of Obstetrics and Gynecology, McMaster University, Hamilton, ON L8N 3Z5 Canada; 5 Faculty of Fisheries, Ege University, 35100 Bornova, İzmir Turkey; 6 Center for Marine Research, Ruđer Bošković Institute, 52210 Rovinj, Croatia

## Abstract

Rare earth elements (REEs) are key constituents of modern technology and play important roles in various chemical and industrial applications. They also are increasingly used in agricultural and zootechnical applications, such as fertilizers and feed additives. Early applications of REEs in agriculture have originated in China over the past several decades with the objective of increasing crop productivity and improving livestock yield (e.g., egg production or piglet growth). Outside China, REE agricultural or zootechnical uses are not currently practiced. A number of peer-reviewed manuscripts have evaluated the adverse and the positive effects of some light REEs (lanthanum and cerium salts) or REE mixtures both in plant growth and in livestock yield. This information was never systematically evaluated from the growing body of scientific literature. The present review was designed to evaluate the available evidence for adverse and/or positive effects of REE exposures in plant and animal biota and the cellular/molecular evidence for the REE-associated effects. The overall information points to shifts from toxic to favorable effects in plant systems at lower REE concentrations (possibly suggesting hormesis). The available evidence for REE use as feed additives may suggest positive outcomes at certain doses but requires further investigations before extending this use for zootechnical purposes.

## Rare Earth Elements: Technological Tools and Matter of Health Concern

An overwhelming number of rare earth elements (REE)-related technological applications have been developed over the last decade, making these elements indispensable to present day life (Rim et al. 2013; Shin et al. 2019). At the same time, another focus of REE toxicity research has been devoted to their multiple adverse effects in a number of biota, as demonstrated in a growing body of literature (Pagano et al. 2015; d’Aquino and Tommasi 2016; Gwenzi et al. 2018). In a number of reports on REE-associated toxicity, biological effect endpoints, such as growth inhibition, cytogenetic anomalies, or redox abnormalities, have been investigated in plant models (d’Aquino et al. 2009a, b; Thomas et al. 2014; Turra 2018). Compared with that extensive body of literature linking REE exposure to adverse effect in plants (> 3000 citations in PubMed), relatively fewer reports have demonstrated positive effects of REEs in promoting plant growth, mainly in field experiments, or in their use as feed additives in livestock. Little information is available on the use of REE-containing fertilizers and their presence and concentrations in soils outside of China. The present review extracted relevant literature linking the keywords “rare earth” with “fertilizer” and “feed additive.” The reviewed literature is based on previously available references and recently retrieved from online databases, including PubMed, Embase, Scopus, Web of Science, and Google Scholar. The selected reports showed a complex, yet suggestive body of evidence focused on the use of REEs as fertilizers and as feed additives.

## REE-Associated Effects on Plant Growth

The effects of specific elements on the growth processes of plants have long been of interest to agronomists and plant physiologists. An early report by Drobkov (1941) demonstrated that the yield of pea plants was increased by the addition of 10^−2^ g lanthanum per vessel. After that pioneering study, the agronomic use of REEs became a growing subject of investigation in subsequent decades (Harmet 1979; Wu et al. 1983). This use was further highlighted in a review by Pang et al. (2002) as an established practice in Chinese agriculture. Recent studies, mostly published in the past decade, have focused on laboratory studies, such as the cellular and molecular effects of REEs on a number of plant species. As summarized in Table 1, published studies were mostly focused on La, Ce, or REE mixtures and their effects on a limited number of plant species. For example, administration of a REE-based fertilizer was shown to increase wheat (*Triticum aestivum*) crop yield and REE accumulation (Zhang and Shan 2001). Furthermore, by administering a REE mixture to wheat plants (*Triticum aestivum*) as foliage-dressing, Liang et al. (2005) found higher REE accumulation in roots and leaves, while no distinct REE residue in grains was found.

**Table 1:** Use of REE-based fertilizers and main observed effects on plant growth, plant organ accumulation and soil microorganisms

Plant species	Observed effects	References
Wheat (*Triticum aestivum*)	Foliage-dressing has a higher accumulation of REEs in root and leaf. No significant accumulation; no residue of REEs in grains was found	Liang et al. (2005)
*Triticum durum*	REE nitrate inhibited seed germination at 0.01 and 0.1 mM, while pre-soaking for 2–4 h inhibited seed germination at higher concentrations	d’Aquino et al. (2009a)
Corn (*Zea mays*) and mungbean (*Vigna radiata*)	Decreased growth or no effects from exposures to La or Ce at levels from 0.2 to 5 mM	Diatloff et al. (2008)
Set of native and crop species	Higher phytotoxicity in native versus crop species in dose–response tests	Thomas et al. (2014) and Carpenter et al. (2015)
Soybean (*Glycine max*)	Low La concentrations stimulated photosynthetic rate and chlorophyll content, leading to a higher incidence of binucleate cells, and to an increase in roots and shoot biomass. At higher La levels, soybean growth was reduced	de Oliveira et al. (2015)
Rice (*Oryza sativa*)	La-associated concentration-dependent modulation of oxidative stress endpoints, consistent with hormetic effects	Xu and Chen (2011) and Liu et al. (2016a, b)
Rice	Ce (25–100 μM) stimulated rice germination and growth	Ramírez-Olvera et al. (2018)
Rice	La positively affects aged seed germination	Fashui et al. (2000)
Rice	Ce positively affects aged seed germination	Fashui (2002)
Rice	La positively affects seed germination and seedling growth	Fashui et al. (2003)
Tea	Cell polysaccharides bound REE sprayied on plants	Wang et al. (2003)
Duckweed (*Lemna minor*)	Ce (0.1 mM) increased growth; 1 mM Ce cau sed oxidative stress	Zicari et al. (2018)
*Allium cepa*	Highest concentration of La and Ce (200 mg/kg) induced significant decrease in root elongation and mitotic index, with increased mitotic aberrations	Kotelnikova et al. (2019)
*Allium cepa*	CeO_2_ microparticles and nanoparticles (12.5–100 ppm) for 4 h had cytotoxic and genotoxic effects	Liman et al. (2019)
Adzuki bean (*Vigna angularis*)	La^3+^ alleviates P-deficiency, improves photosynthesis and decreases oxidative stress	Lian et al. (2019)
*Trichoderma* strains	Tolerance to La^3+^ and REE mixture, and dose-dependent effects	d’Aquino et al. (2009b)
*Trichoderma viride*, *T. hartianum*	La and REE mixture induce inhibitory effects in liquid cultures	Tang et al. (2004)

Major REEs, such as La and Ce, can affect uptake and accumulation of nutrient elements, and high concentrations alone or in combination can inhibit root elongation of wheat (Hu et al. 2002). d’Aquino et al. (2009a) treated durum wheat (*Triticum durum* Desf.) with a REE mixture and found inhibition of seed germination and decrease of plantlet growth at 0.01–0.1 mM. Higher La and Ce levels (0.2–5 mM) were tested in *Zea mays* and *Vigna radiata* by Diatloff et al. (2008) who found that at 1 and 5 mM, La and Ce significantly decreased the shoot dry weight of corn and mungbean. The results showed that germination was inhibited by Ce at low pH, with reductions in biomass for the two native forb species after exposure to all REEs. Moreover, root biomass of native species was affected at lower doses than in crop species. REE uptake by plants was higher in the below-ground parts than in the above-ground plant tissues. In corn (*Zea mays*), REE accumulation was observed in roots and improved P-uptake and crop yield (Xu and Wang 2007; Emmanuel et al. 2010). The changes in crop production were explained in part by REE-induced changes to antioxidant activity (i.e., peroxidase and superoxide dismutase activity) (Emmanuel et al. 2010). de Oliveira et al. (2015) exposed soybean plants to La (5–160 µM) and found that low La concentrations stimulated photosynthetic rate and chlorophyll content, leading to a higher incidence of binucleate cells and to an increase in root and shoot biomass. At higher La levels, soybean growth was reduced. Indeed, exposure to REEs, particularly La-containing REEs, significantly impacts plant growth and nutrient quality (von Tucher and Schmidhalter 2005; Xiong et al. 2006; Wang et al. 2012; Ren et al. 2016). Wang et al. (2012) found evidence that these effects may be due to DNA breaks and DNA-protein crosslings. Furthermore, it was suggested that REEs can activate endocytosis in plant cells and facilitate REE deposition (Wang et al. 2014). Two other studies (Xu and Chen 2011; Liu et al. 2016a, b) tested the effects of La^3+^ (0.05–1.5 mM) on reactive oxygen species (ROS) production and antioxidant metabolism in the roots of rice (*Oryza sativa*). ROS levels declined after treatment with 0.05 mM La^3+^, and antioxidant metabolism was enhanced. Similar results were found by exposing rice or duckweed to Ce, with an evident shift from stimulating to inhibitory effects by increasing Ce concentrations (Ramírez-Olvera et al. 2018; Zicari et al. 2018). While the effects of Ce may be species-specific, as measured in eight different plant species, soil properties also can significantly influence the severity of Ce phytotoxicity (Moreira et al. 2019).

A recent report by Kotelnikova et al. (2019) evaluated La and Ce toxicity on onion (*Allium cepa*) in solutions and amended to soil. The authors found a significant decrease in root elongation and mitotic index, with increased mitotic aberrations, and significant cytotoxicity of soil samples containing the highest La and Ce concentration (200 mg/kg). Another recent study of REE-associated toxicity in *Allium cepa* reported that CeO_2_ microparticles and nanoparticles (12.5–100 ppm) exerted cytotoxic and genotoxic effects (Liman et al. 2019). In fact, there are reports of CeO_2_ and other REE (La_2_O_3_, Gd_2_O_3_, Yb_2_O_3_) nanoparticles inhibiting root elongation in several crop species (Ma et al. 2010). Adzuki bean (*Vigna angularis*) seedlings were exposed to LaNO_3_ (150 mg/L), and changes in growth, photosynthetic ability, and phosphorus-use efficiency under P-deficiency conditions were concurrently evaluated (Lian et al. 2019). The negative effects of P-deficiency on photosynthetic activity and chlorophyll content in leaves were alleviated by La^3+^ treatment. In turn, phosphate fertilization and liming were considered to be significant sources of REEs and soils receiving continuously high doses of these inputs are likely to be enriched in REEs (Silva et al. 2019) at concentrations that should be considered under risk assessment frameworks.

REE-associated effects on field trials are very limited. Some data concern the effects of spraying REE fertilizer on tea (Wang et al. 2003). The results of field experiments showed the REE concentrations in new shoots of tea plants and REE concentrations in soil were significantly correlated. Approximately 10% of soluble REEs in tea infusion was bound to polysaccharide, and the amount of REE-bound polysaccharide decreased over time. At least a 25-day safety interval was needed between spraying and harvesting if the microelement fertilizer was used to enhance tea output and to ensure consumption safety.

Little information is available on the effect of REEs on soil microbiota and the role of microbiota in the balance between the chemical forms and bioavailability of REEs in soil, which may affect their uptake and distribution in plant tissues, as REE mixtures and La affect the growth of some bacteria and fungi (d’Aquino and Tommasi 2016). Soil-borne fungi (*Trichoderma atroviride*, *T. harzianum*, *Botrytis cinerea*, *Alternaria alternata*, *Fusarium solani*, *Rhizoctonia solani*, and *Sclerotinia sclerotiorum*) were found to display an overall good tolerance to the presence of several REEs in the culture medium (d’Aquino et al. 2009b). Growth inhibitory effects were detected in plate tests when La or a REE mixture (containing La, Ce, Pr, Nd, and Gd) were supplied at concentrations higher than 100 mM. In liquid culture tests, inhibitory effects were observed on the growth of *T. atroviride* and *T. harzianum* when exposed to La and REE mixture at concentrations from 1 to 10 mM (Tang et al. 2004). In this study, REE accumulation also had stimulatory effects with concurrent inhibition and restimulation of soil bacteria and actinomycetes, along with a continuous stimulation on soil fungi. The interactions between REEs and microbial cells were influenced by many parameters, such as cell structure and external conditions, as well as ionic availability attesting to the complex inter-relationships of REEs with many ecosystem components (d’Aquino et al. 2009b).

The effects of soil REEs on navel orange quality and safety were investigated by Cheng et al. (2015) in REE mining areas. This study investigated the transfer characteristics of REEs from soil to navel orange pulp (*Citrus sinensis* Osbeck cv. Newhall) and the effects of soil REE content on the internal fruit quality at concentrations ranging from 38.6 to 546 mg/kg. The REE content in the main organs of the navel orange decreased in the following order: root > leaf > peel > pulp. The REE accumulation capacity of navel orange pulp is relatively low, and the REE content in pulp was approximately 14 times lower than the food safety limit set in China (Cheng et al. 2015).

The distribution of 16 REEs (Sc, Y, and 14 lanthanoid elements) in field-grown maize and the concentration of heavy metals in the grains after application of REE-containing fertilizers were studied in treated crops during the vegetation growth stage (Xu et al. 2002). Ten days after REE administration, significant dose-dependent accumulative effects of individual REEs in maize roots and shoots were observed, except for Sc and Lu (Xu et al. 2002). At the 2 kg/ha REE fertilizer levels, accumulative concentrations of light REEs (La, Ce, Pr, and Nd) and Gd in the plant shoots were significantly higher than in controls. Concentrations of individual REEs in field-grown maize after REE administration decreased in the order of root > leaf > stem > grain. During the vegetative growth period, selective accumulation of individual REEs (La, Ce) in the roots were found in a dynamic equilibrium, and the distribution of these elements in the plants was variable. At a dosage of less than 10 kg/ha REEs, no accumulative concentrations of individual REEs were detected in maize grains. Under the experimental conditions, the application of REE-containing fertilizers did not induce increases in REE concentrations in grains. The authors concluded that the REE dosage currently applied in China (0.23 kg/ha/year) can hardly affect the safety of maize grains in arable soils, even over a long period (Xu et al. 2002).

Data concerning the effects of REEs on seed germination are still contradictory. Thomas et al. (2014) reported on the effect of La, Y, and Ce on seed germination in selected crop and wild plant species. La and Ce contamination at high pH had no impact on seed germination in the tested species at any dose, whereas Ce supplied at low pH induced negative effects on seed germination in *Asclepias syriaca*, *Panicum virgatum*, *Raphanus sativus*, and *Solanum lycopersicum*. Yttrium severely affected seed germination in *Desmodium canadense* and *S. lycopersicum.* The authors suggested that the slow accumulation rate of REEs in the environment could be problematic, even if limited effects have so far been reported on seed germination in different species. Other data reported positive effects of La on germination of aged rice seeds (Fashui 2002; Fashui et al. 2000, 2003). On the other hand, exposure to different REEs in the soil had no effects on the germination of many wild and crop plant species; only Nd and Er reduced germination in *Raphanus sativus* and in tomato, respectively. Not clear were the effects of Pr and Sm, which induced negative effects at low but not at high concentrations (Carpenter et al. 2015). A recent review by Agathokleous et al. (2019) focused on REE-associated hormesis, i.e., stimulation of several biological functions exerted by low levels of agents inducing inhibition (toxicity) at higher concentrations. This report critically evaluated the body of literature assessing the hormetic effects of La on plant physiology and found substantial evidence for La-associated hormesis. Following the pioneering study by Stebbing (1982), an extensive number of reports demonstrated the occurrence of hormesis deriving from the effects of a number of physical and chemical agents, including metals (Poschenrieder et al. 2013; Morkunas et al. 2018) and, more specifically, REEs (Nascarella and Calabrese 2016; Liu et al. 2016a, b; Xu and Chen 2011; Ramírez-Olvera et al. 2018; Zicari et al. 2018; Oliveira Duarte et al. 2018). Altogether, the present knowledge of La- and REE-associated hormesis may provide grounds for supporting the use of REEs—at appropriate, growth enhancing concentrations—as fertilizers to boost crop yields, beyond Chinese agriculture, in wider geographical regions. Decision-making should rely on appropriate investigations confirming the lack of REE accumulation in the edible portions of plants that could inadvertently lead to negative health outcomes to secondary consumers (Xu et al. 2002; Liang et al. 2005). Beyond hormesis, REEs have been investigated as essential elements involved in basic biological events, thus raising their biological significance to a number of different biota (Daumann 2019).

Other lines of evidence focused on the REE-associated interferences with adverse events or improvements of plant growth or quality. This was the case for Ce-induced growth enhancement of tomato plants by counteracting *Fusarium* wilt infection (Adisa et al. 2018, 2020). A complementary finding also was reported by Liang et al. (2006) who found Ce-associated protection of soybean seedlings when exposed to two levels of supplementary UV-B radiation. A study by Ma et al. (2014) evaluated the effects of spray application of La and Ce solutions on yield and quality of Chinese cabbage (*Brassica chinensis*) in different seasons. These authors found that treatments in spring and autumn promoted plant growth, with increased fresh and dry weight ratios of harvested material. Together, the weight of evidence suggests that at appropriate concentrations, positive outcomes in plant growth can be achieved with minimal risk to secondary consumers who eat the fruits and/or grain. As a final consideration, Ren et al. (2016) reported that REE-based fertilizers increased plant growth in both spring and autumn; however, increases for Chinese cabbage were greater in the autumn than in the spring while the opposite was seen for turnips (*Brassica napus*). Therefore, crop species and growing season should be considered when applying REE fertilizers to crops (Ren et al. 2016).

## REEs as Feed Additives in Livestock

The use of REE preparations as additives in livestock feed has been practiced for several decades (Ji and Cui 1988; Xu et al. 1999) as reviewed by Rambeck and Wehr (2005) and by Redling (2006). Recently, the use of REE-based feed additives were reviewed by Squadrone et al. (2018), Abdelnour et al. (2019), and Tariq et al. (2020). Another review by Flachowsky et al. (2019) raised substantial doubts on the effectiveness of REE supplementation in feed additives. The main goals of these zootechnical procedures in poultry were focused on egg yield and improved egg quality by administering REE feed additives to laying hens. Increased growth rate in REE-supplemented chicks, piglets, or ruminants also were sought and discussed by the EFSA Panel on Additives and Products of Substances used in Animal Feed (FEEDAP) (EFSA 2016, 2019) and are summarized in Table 2. The European Commission just enacted the law No. 2020/1370 on October 1, 2020 approving the use of REE citrate “Lancer” feed additive for weaned piglets (Official Journal of the European Union 2020). Before this EC act, an established body of literature had pointed to the use of REE-based feed additives in poultry and pig breeding.

**Table 2:** Use of REE-based feed additives in livestock: observed effects

Target species/strains	Tested REEs	REE concentration (mg/kg)	Observed effects	References
1. Poultry				
Ross broiler chicks	REE citrate or REE chloride	70	Increased gain in body weight and feed conversion ratio	He et al. (2010)
Arbor Acre broiler chicks	LaCl_3_ or La_2_O_3_	100–400	No significant difference in the final live weight, daily weight gain and feed conversion ratio	Igbasan and Adebayo (2012)
Ross 308 1d-old broilers	REE-enriched yeast	500–1500	No significant influence on growth performance, but improved nutrient digestibility and meat quality	Cai et al. (2015)
Lohman LSL laying hens	La_2_O_3_	100–400	Significantly increased egg production; no effect on egg weight; decreased MDA and TBARS	Durmuş and Bölükbaşı (2015)
Lohman LSL laying hens	CeO_2_	100–400	Increased egg production, feed conversion ratio and egg shelf life; decreased SOD, MDA and TBARS	Bölükbaşı et al. (2016)
ISA brown laying hens	REE-enriched yeast	500–1000	Significantly increased egg production and coefficient of apparent digestibility	Cai et al. (2016)
Japanese quails	REE citrate	50–200	Improved growth and efficiency of protein and energy utilization	Eleraky and Rambeck (2011)
2. Pigs				
Piglets	La_2_O_3_ or REE mixture	300	Gain in body weight increase and feed conversion ratio	He and Rambeck (2000) and He et al. (2001)
(Landrace × Yorkshire) × Duroc finishing pigs	REE-enriched yeast	500–1500	Improved growth performance, digestibility, blood lymphocyte counts, and fecal Lactobacillus counts	Cai et al. (2018)
Piglets	REE mixture	200	Growth performance of REE-citrate and control fed piglets did not differ significantly	Kraatz et al. (2006)
Sows and piglets	REE mixture	200	Improved antioxidant effects and immunity of sows and piglets	Xiong et al. (2019)
3. Ruminants				
West African dwarf sheep	La_2_O_3_	100–300	Better daily weight gain and total weight gain; no significant effects on serum and hematological endpoints, except for significant WBC increase	Adu et al. (2006)
German Holstein bulls	REE citrate	100–300	Decreased weight gain; no increase in nutrient digestibility	Schwabe et al. (2011)
Ruminally cannulated Simmental steers	LaCl_3_	450–1800	Improved rumen fermentation and feed digestion; simulated digestive microorganisms or enzymes	Liu et al. (2008)
Ruminally cannulated Simmental steers	CeCl_3_	80–240	Increased the digestibility of neutral detergent fibre, decreased molar ratio of rumen acetate to propionate	Lin et al. (2015)

Broiler chicks were tested for growth and feed conversion ratio after administration of REE feed supplements in five independent studies. He et al. (2010) tested the effects of REE citrate or REE chloride at a concentration of 70 mg/kg and found increased gain in body weight and feed conversion ratio. Similarly, Agbede et al. (2011) found that La_2_O_3_ (100–300 mg/kg) increased total weight gain and altered heart, spleen, and liver weights. Furthermore, REE supplementation led to changes in hematological and nutritional parameters, including total red blood cell count, hemoglobin and differential leukocytes, cholesterol, and bicarbonate (Agbede et al. 2011; Ozung et al. 2019). Two studies (Igbasan and Adebayo 2012; Cai et al. 2015) failed to find increased gain in chick body weight by administering La or Ce or REE mixtures as feed additives at concentrations ranging from 100 to 1500 mg/kg. These apparently contradictory data might suggest that lower REE doses might be more effective in promoting growth gain than the higher REE concentrations utilized in the latter two studies (He et al. 2001; Igbasan and Adebayo 2012; Cai et al. 2015).

The effects of REE-based feed supplements on laying hens were investigated in terms of egg production and egg quality. A series of studies consistently reported an increase in egg production, along with a decrease in oxidative stress endpoints following administration of La or Ce oxides or REE mixtures at concentrations ranging from 100 to 1000 mg/kg (Durmuş and Bölükbaşı 2015; Bölükbaşı et al. 2016; Cai et al. 2016). Bölükbaşı et al. (2016) found that low-dose of Ce oxide addition significantly increased serum calcium and phosphorus, paralleling what was found by Reka et al. (2018) in White Leghorn layers. Moreover, feed conversion ratio, egg shelf life, and coefficient of apparent digestibility were found to be increased following REE supplementation. In a related species, Eleraky and Rambeck (2011) tested REE supplementation on growth performance of Japanese quails and found increased weight gain compared with control group with improved feed conversion ratio and efficiency of protein and energy utilization.

Administration of REE-supplemented pig feeds were evaluated in a series of studies in terms of growth performance, feed conversion ratio, and digestibility. Early studies testing supplementation with higher REE dosages (300 mg/kg) reported on increased piglet growth performance (He and Rambeck 2000; He et al. 2001; Schuller et al. 2002; Wang and Xu 2003). This growth-promoting effect of REE- enriched yeast was confirmed by Cai et al. (2018). However, a previous report by Kraatz et al. (2006) failed to find increased growth performance of pigs following feed supplementation with 200 mg/kg of REE mixture. Furthermore, Förster et al. (2008) found that supplementation with a REE mixture decreased feed intake but increased thyroid hormone levels in rearing piglets. A recent report by Xiong et al. (2019) tested immunity and antioxidant activity in sows and their piglets supplemented with a REE mixture (200 mg/kg) and found improved antioxidant and immunity both in sows and in piglets.

A more limited body of work focused on the effects of REE feed addition in ruminants. Adu et al. (2006) reported increased weight gain in West African dwarf sheep supplemented with La_2_O_3_ (100–300 mg/kg), without significant effects on serum and hematological endpoints. Schwabe et al. (2011, 2012) supplemented German Holstein bulls with REE citrate (100–300 mg/kg) finding decreased weight gain, but accumulation in liver, kidneys, and rib bone with increasing REE supplementation. Three studies were conducted on ruminally cannulated steers and found improved rumen fermentation and increased digestibility of neutral detergent fiber (Liu et al. 2008; Xun et al. 2014; Lin et al. 2015).

## REE-Associated Uses in Agriculture and Zootechny: State-of-the-Art and Prospects

The body of literature on the uses of REEs as fertilizers and as feed additives in livestock suggests the following outcomes:

1. REE-associated effects in plant models ranged from growth stimulation (hormesis) to inhibitory effects as a function of REE concentration (Poschenrieder 2013; Morkunas et al. 2018; Nascarella and Calabrese 2016; Liu et al. 2016a, b; Xu et al. 2002);
2. Different accumulation gradients were noted in plant and animal organs, with minimal—if any—accumulation in the edible parts (Xu et al. 2002; Tariq et al. 2020);
3. REE-based feed additives in poultry failed to result in increased weight gain in broiler chicks, whereas REE-supplemented laying hens improved quantitative and qualitative egg production (Durmuş and Bölükbaşı 2015; Bölükbaşı et al. 2016; Cai et al. 2016);
4. Growth performance in REE-supplemented piglets appeared to depend on REE dosages, as increased weight gain was observed for REE dosages > 300 mg/kg, and not for a dosage of 200 mg/kg (He and Rambeck 2000; He et al. 2001);
5. Little, if any, conclusion can be drawn from reports on REE supplementation in ruminants, except for a study of REE feed additive in sheep, resulting in increased weight gain. The published reports focused on cattle and on changes, if any, to rumen function and content.

Altogether, the practices of amending soils or supplementing animal diet with REE, well established in the Chinese agronomic and zootechnical spheres, appear to offer suggestive and stimulating challenges worldwide in terms of achieving improved crop or livestock yield. Rare earth elements could be a potentially important breakthrough in agronomy in a world with increasing food security issues. Further investigations and qualified decision-making based on the presently available and research data are warranted.
